# Rhino Conjunctivitis and Asthma Among Seafood Processing Workers in Greenland. A Cross-Sectional Study

**DOI:** 10.3389/falgy.2021.747011

**Published:** 2021-10-08

**Authors:** Birgitte Hamann Laustsen, Øyvind Omland, Else Toft Würtz, Torben Sigsgaard, Niels E. Ebbehøj, Ole Carstensen, Kurt Rasmussen, Sandip D. Kamath, Andreas L. Lopata, Jakob Hjort Bønløkke

**Affiliations:** ^1^Department of Clinical Medicine, Danish Ramazzini Centre, Aalborg University, Aalborg, Denmark; ^2^Institute of Nursing & Health Science, Ilisimatusarfik, University of Greenland, Nuuk, Greenland; ^3^Department of Clinical Medicine, Aalborg University, Aalborg, Denmark; ^4^Department of Occupational Medicine, Danish Ramazzini Centre, Aarhus University Hospital, Aarhus, Denmark; ^5^Department of Public Health, Aarhus University, Aarhus, Denmark; ^6^Department of Occupational and Environmental Medicine, Bispebjerg Hospital, Copenhagen, Denmark; ^7^Department of Occupational Medicine, Danish Ramazzini Center, Regional Hospital West Jutland, Herning, Denmark; ^8^Molecular Allergy Research Laboratory, College of Public Health, Medical and Veterinary Sciences, Australian Institute of Tropical Health and Medicine, James Cook University, Townsville, QLD, Australia; ^9^Department of Occupational and Environmental Medicine, Danish Ramazzini Centre, Aalborg University Hospital, Aalborg, Denmark

**Keywords:** occupational exposure, asthma, allergy, seafood, snow crab, shrimp, Anisakis, Greenland

## Abstract

**Introduction:** The fishing- and the seafood processing industries are the largest industrial sectors in Greenland. Despite this, only a few cases of occupational diseases in this industry have been reported to the Danish Labor Market Insurance. Occupational asthma and allergy are well-known occupational diseases in the seafood processing industry worldwide and underreporting of occupational diseases in Greenland is suspected.

**Objective:** The aim of the current study was to examine the associations between job exposures and occupational asthma and rhino conjunctivitis in workers in the Greenlandic seafood processing industry and to compare the prevalence of sensitization by type and degree of exposure to snow crab, shrimp, fish, and the fish parasite, *Anisakis simplex*.

**Methods:** Data from 382 Greenlandic seafood processing workers were collected during 2016–2018. Data included questionnaire answers, lung function measurements, skin prick tests, and blood samples with ImmunoCAP. For all analyses, *p* < 0.05 was considered the level of significance.

**Results:** 5.5% of the workers had occupational asthma and 4.6% had occupational rhino conjunctivitis. A large proportion of the workers were sensitized to allergens specific to the workplace; 18.1% to snow crab, 13.6% to shrimp, 1.4% to fish, and 32.6% to the fish parasite, *A. simplex*. We found a dose-response relationship between the risk of being sensitized to snow crab and *A. simplex* and years of exposure to the allergens in the seafood processing industry.

**Conclusion:** This study showed that a considerable proportion of workers in the Greenlandic seafood processing industry had occupational asthma and rhino conjunctivitis. Additionally, the study showed high sensitization levels toward snow crab, shrimp, and the fish parasite, *A. simplex*. This supports the hypothesis of a considerable degree of underreporting of occupational allergic airway disease in the Greenlandic seafood processing industry. Prospectively, it is important to inform workers, leaders, and health care professionals of the health problems and the law on worker's compensation, and to initiate preventive actions at factory and trawler level.

## Introduction

The fishing- and the seafood processing industries are the largest industrial sectors in Greenland. In 2018, these sectors occupied approximately 16% of the Greenlandic workforce of approximately 26.800 people (1). High sensitization rates toward snow crab and shrimp have been found in Canadian studies as well as a high prevalence of both occupational asthma and occupational allergy (symptoms at work from the upper airways in combination with sensitization to seafood) in the seafood processing industry (2–4). Overall, the studies have shown an occupational asthma prevalence of 2–36% and an occupational allergy prevalence of 5–24% in the seafood processing industry (5, 6). A dose–response relationship between the prevalence of occupational asthma and the level of exposure to seafood aeroallergens has been established, showing a higher prevalence of asthma with higher exposure levels (5). It has been known for decades that the mechanism for sensitization toward airborne seafood allergens at work is IgE antibody mediated (2, 7). Later studies have shown that especially high molecular weight proteins are involved in the pathogenesis (8). In addition to identified allergens from the seafood itself, e.g., parvalbumin and tropomyosin, also allergens from parasites such as the fish parasite, *Anisakis simplex (A. simplex)*, can induce allergic sensitization (9). The adult *A. simplex* larvae live in the digestive system of marine mammals from which their eggs are expelled to the sea by feces. The eggs evolve through several steps and are infectious as third-stage larvae in the tissue of fish (10). When fish are eaten by humans, we are at risk of becoming an accidental host of the parasite larvae causing Anisakiasis, a gastro-intestinal disease. *A. simplex* can also cause allergic reactions in humans with symptoms such as urticaria. Infection with live larvae are meant to be a prerequisite to sensitization but dead larvae can also lead to allergic reactions such as asthma, rhino conjunctivitis and dermatitis when inhaled or by direct contact (9, 11, 12). Sensitization levels toward *A. simplex* in the seafood processing industries are shown to vary from 1.8 to 50% (13, 14).

Knowledge regarding occupational asthma and allergy in Greenland is limited since only one pilot study has addressed this subject, reporting a 40% IgE sensitization to snow crab, 20% sensitization to shrimp and 10% sensitization to cod (6). Sensitization levels toward *A. simplex* were 26%. Workers suffering from probable occupational asthma constituted 11%, while workers suffering from possible occupational asthma constituted 20%. The study indicates that occupational asthma and allergy might represent a serious problem in the Greenlandic seafood processing industry. However, despite the size of the Greenlandic seafood processing industry, only two cases of occupational diseases concerning “agriculture, forestry and fishing industry” were reported to the Danish Labor Market Insurance in 2014 (15). The diagnoses reported correspond poorly to what one would expect given the occupational structure in Greenland with asthma and allergy representing <10% in 2014 (15). A considerable degree of underreporting is therefore suspected. Alternatively, the population of Greenland, of which most are of Inuit origin, could be suspected of being partly genetically protected against developing respiratory diseases (16, 17). This could partly explain why so few occupational respiratory diseases are reported.

The aim of the current study was to examine the associations between job exposures and occupational asthma and rhino conjunctivitis in workers in the Greenlandic seafood processing industry and to compare the prevalence of sensitization by type and degree of exposure to snow crab, shrimp, fish, and the fish parasite, *A. simplex*.

## Materials and Methods

### Study Population

The study was conducted at the west coast of Greenland from 2016 to 2018. A total of 382 workers participated. They were employed at three large factories with 60–118 employees located in the largest cities in Greenland, Nuuk, Sisimiut, and Ilulissat, four smaller factories with 12–40 employees located in four small settlements, and four factory trawlers with 11–34 employees. All employees were invited to participate, but the number of eligible workers could not be precisely determined because several were seasonal workers or were in the process of being hired or leaving the jobs. All workers that accepted the invitation were included even if they were seasonal workers. From employment lists and talks with the leaders, we estimated that approximately 85% of the invited employees participated in the study. Of the employees present on the examination day, 99% participated. The study was approved by The Danish Data Protection Agency, the Central Denmark Region, The Scientific Ethical Committee for Greenland (2015-11317) and the Human Research Ethics Committee for James Cook University, Australia (H8114). Written and oral, informed consent was obtained from each participant.

### Data Collection

Data were collected in 2016–2018 by a team of doctors specialized in occupational medicine. At the workplace the participants received a questionnaire and underwent clinical examinations with spirometry, skin prick tests, and venous blood samples. A modified version of the ECRHS II lung function questionnaire^1^ was translated into Danish and Greenlandic. It included questions regarding ethnicity, work exposures, health, smoking, and diet. Clinical interviews were performed with the aid of native medical students, who were fluent in Danish and Greenlandic language, if the participants were unable to fill in the questionnaires themselves.

Spirometry was performed using a spirometer (MIR Spirobank II spirometer and Easy-One^®^ NDD Medical Technologies, Zurich, Switzerland). Lung function was registered as forced expiratory volume in the first second of expiration (FEV_1_) and forced vital capacity (FVC) as best out of at least 3 blows. A post bronchodilator test was performed 15 min after inhalation of 0.2 mg Salbutamol if FEV_1_/FVC was reduced by 20% or more in proportion to expected. The bronchodilator test was deemed positive if lung function measured by FEV_1_ and/or FVC showed improvement >12% and at least 200 ml increase after inhalation of β_2_-agonist (18). Additionally, 30 participants were asked to observe their lung function for 2 weeks by peak-flow monitoring. Due to lack of compliance, these measurements were not included in the analyses. Using gender to represent biological sex, predicted values, and z-scores for spirometric indices were calculated using the Quanjer GLI-2012 regression equations for Caucasians by means of the SPSS macro provided by the E.G.L.F. Initiative^2^ (19).

Skin prick tests were performed using a standard prick test panel (Soluprick, ALK-Abelló, Hørsholm, Denmark), including birch, grass mix, mug wort, horse, dog, cat, house-dust mite, and mold. Additionally, in-house generated seafood preparations were used, including extracts from Snow-crab (*Chionoecetes opilio*) (minced entire crab with entrails, shell, mouth, etc., raw meat, cooked meat, cooking water), Northern prawn (*Pandalus borealis*) (raw meat, cooked meat, cooking water), Greenland cod (*Gadus ogac*) (raw meat), and Greenland turbot (*Reinhardtius hippoglossoides*) (raw meat). A wheal diameter of ≥3 mm with a positive reaction to histamine phosphate (1 mg/ml) and negative to saline was considered as a positive test result.

Serum samples were analyzed at the Molecular Allergy Research Laboratory, Australian Institute of Tropical Health and Medicine, James Cook University, Australia. Sera were assayed for IgE-specific antibodies to cod (f3) and *A. simplex* (p4) by ImmunoCAP (Thermo Fisher Scientific, Waltham, Mass, USA). The test was considered positive if IgE antibody readout was >0.10 kU/L.

### Exposure

The snow crab process included manual slaughtering, cooking, freezing, and packing. The shrimp process included landing, maturation in big vessels, boiling, manual, and mechanical removal of the shell and packing. The fish process including landing, decapitation and degutting, fileting, freezing, and packing. Crab and fish processing are seasonal activities and the study was conducted during the processing season. Shrimp is processed all year.

Current employment was comprised by the total number of years the participants had been employed in the current facility. Participants were exposed to snow crab in four different work tasks including crab handling, cold store work, cleaning, and laboratory work. They were exposed to shrimp in six different work tasks including shrimp handling, cold store work, cleaning, laboratory work, and other work in the production site and the hold. Finally, the participants were exposed to fish in six different work tasks including fish handling, cold store work, cleaning, laboratory work, and other work in the production site and the hold. Exposure to snow crab, shrimp, and fish was calculated based on the number of years employed in the current facility times the average time spent in each job function with exposure to snow crab, shrimp, or fish. *A. simplex* antigen exposure was suspected to be most common during fish processing and calculated as number of years working with fish processing.

Former employment was assessed depending on the total number of years exposed to fish, shrimp, and/or crab in the participant's former employments.

### Classification of Occupational Asthma

Participants were classified with occupational asthma into four categories according to Gautrin et al. (4):

- Probable occupational asthma: Participants presenting at least one symptom from the lower airways at work (cough, shortness of breath, wheezing) with improvement of symptoms outside work or a positive reversibility test and sensitization to at least one work allergen toward snow crab, shrimp, or fish.
- Possible occupational asthma: Participants reporting at least one symptom from the lower airways at work, improvement of symptoms outside work but no sensitization toward work allergens.
- Unlikely occupational asthma: Participants reporting no symptoms from the lower airways at work but sensitization to at least one work allergen.
- Negative (no occupational asthma): Participants reporting no symptoms from the lower airways at work and no sensitization to work allergens.

### Classification of Occupational Rhino Conjunctivitis

Participants were classified with occupational rhino conjunctivitis into four categories according to Gautrin et al. (4):

- Probable occupational rhino conjunctivitis: Participants reporting at least one symptom from the upper airways at work (runny nose, sneezing, irritation in eyes, and/or throat) and sensitization to at least one work allergen.
- Possible occupational rhino conjunctivitis: Participant reporting at least one symptom from the upper airways at work and at least one symptom from the lower airways at work (cough, shortness of breath, wheezing) but no sensitization to any work allergen.
- Unlikely occupational rhino conjunctivitis: Participants reporting at least one symptom from the upper airways at work but no symptoms from the lower airways at work and no sensitization to any work allergen.
- Negative (no occupational rhino conjunctivitis): participants reporting no symptoms from upper airways at work regardless of sensitization to work allergens.

### Statistical Analyses

Sex differences in health outcome and exposure were tested by *t*-test if the dependent variables were continuous and normally distributed. If the dependent variables were not normally distributed, Mann-Whitney U-test was used. For categorical variables, Pearson Chi^2^-test or Fisher's exact test were used depending on sample size. Associations between duration of exposure to seafood and health outcome were tested with logistic regression with test for trend using post-estimation. Test for trend with stratification were performed using nptrend test. Statistical analyses were performed with Stata version 15.1 (StataCorp LLC, College Station, Texas). For all analyses, *p* < 0.05 was considered the level of significance. IBM SPSS Statistics version 25 was used for the predicted lung function values calculations^3^.

## Results

### Study Population

The study population was composed of 382 workers of which 276 were males and 106 females. Most workers (*n* = 352) identified themselves as Inuit. The remaining 30 participants were from Denmark (*n* = 17) followed by workers from the Faroe Islands, Philippines, Germany, Nigeria, and Iran. There was no difference in age between males and females, with a mean age of 39 years. The proportion of current smokers (*n* = 266) was similar among males and females. The majority (*n* = 250) of both males and females worked at large factories. More males than females worked on trawlers (*n* = 69) (Table 1). A large proportion of the participants were exposed to work allergens both at their current and former workplace with 262 ever exposed to shrimp followed by 242 to fish and 148 to snow crab (Table 2). Only males were engaged in eight of 18 work tasks, primarily loading, repairment, engine room work, and cold store work. More males than females were engaged in service and landing of fish, shrimps, and crabs. More females than males were engaged in fish and shrimp handling and work at the laboratory, where they were mostly involved in food safety procedures of material sampled at the production sites. An equal proportion of males and females were engaged in crab handling, cleaning, office work, and packing.

**Table 1:** Characteristics of the study population.

**Variable**	**Male (♂) *N* = 276**	**Female (♀) *N* = 106**	**Total *N* = 382**	***P*-value for male-female difference**
Age, mean [SD] years	39.6 [13.8]	38.1 [13.1]	39.2 [13.6]	0.34^a^
Age range, years	16.0–68.1	16.9–64.0	16.0–68.1	–
Smoking, %				0.98^b^
Yes	69.8	70.5	70.0	
Never	10.2	10.5	10.3	
Previous	20.0	19.1	19.7	
Pack years among smokers and previous smokers, mean [SD] years	12.5 [14.3]	8.5 [7.4]	11.4 [12.9]	0.09^c^
Ethnicity, % Inuit/other	91.6/8.4	94.3/5.7	92.4/7.6	0.29^b^
Workplace, %				** <0.01** ^b^
Large factory	62.0	74.5	65.4	
Trawler	24.3	1.9	18.1	
Small factory	13.8	23.6	16.5	

a *Independent samples t-test*.

b *Pearson Chi^2^-test*.

c *Mann-Whitney U-test. Bold value indicates p < 0.05*.

**Table 2:** Exposure characteristics of the study population.

**Variable**	**Male (♂)**	**Female (♀)**	**Total**	***P*-value for male-female difference**
Exposed to snow crab^c^, *n* (%)				**0.01^a^**
Currently	49 (18.3)	22 (21.6)	71 (19.2)	
Previously	67 (25.0)	10 (9.8)	77 (20.8)	
Never	152 (56.7)	70 (68.6)	222 (60.0)	
Exposed to shrimp^c^, *n* (%)				**<0.01^a^**
Currently	89 (33.1)	57 (55.9)	146 (39.4)	
Previously	101 (37.6)	15 (14.7)	116 (31.3)	
Never	79 (29.4)	30 (29.4)	109 (29.4)	
Exposed to fish^c^, *n* (%)				0.09^a^
Currently	43 (16.0)	16 (15.7)	59 (15.0)	
Previously	141 (52.6)	42 (41.2)	183 (49.5)	
Never	84 (31.3)	44 (43.1)	128 (34.6)	
Total exposure to snow-crab, mean [SD] years	1.4 [2.9]	1.0 [3.2]	1.3 [2.0]	0.33^b^
Total exposure to shrimp, mean [SD] years	4.6 [7.2]	4.6 [8.1]	4.6 [7.5]	0.97^b^
Total exposure to fish, mean [SD] years	3.5 [6.1]	2.4 [5.3]	3.2 [5.9]	0.09^b^

a *Pearson Chi^2^-test*.

b *Independent samples t-test*.

c *Of participants exposed to snow crab, 12 were missing, to shrimp, 11 were missing, and to fish, 12 were missing. Missings were due to lacking information in the questionnaire regarding work tasks or years of employment. Bold value indicates p < 0.05*.

### Clinical Investigations

Participants with atopy based on skin prick test toward common aeroallergens constituted 23.2% in total (Table 3). A higher proportion was sensitized toward snow crab among atopics (35.4%) than among non-atopics (12.9%) (*p* < 0.01). No difference in the proportion sensitized toward shrimp, fish, or Anisakis was found among atopics vs. non-atopics. Of all the participants, 54 (14.3%) reported earlier symptoms of hay fever (sneezing, runny/itching nose, runny/itching eyes during specific seasons of the year) and 33 (8.7%) reported an earlier diagnosis of asthma by a doctor. Only six participants reported to be medically treated for asthma. Of these six participants, one was classified with probable occupational asthma and three were classified with negative occupational asthma. Possible workplace sensitization included *A. simplex* (32.6%) followed by snow crab (18.1%), shrimp (13.6%), and fish (1.4%) with no sex difference (Table 3). Overall, lung function was alike among males and females, though FVC was just significantly lower among females. In general, the mean FEV_1_ and FVC assessed by z-score were larger than zero (Table 3). Of all the participants, 6% had a FEV_1_ z-score below −1.645, 17% had a FEV_1_ z-score above 1.645, 8% had a FVC z-score below −1.645 and 21% had a FVC z-score above 1.645. Correcting FEV_1_ z-score and FVC z-score with the ratio 0.54/0.52 due to genetical differences among Caucasians and Inuit led to similar FEV_1_ z-score and FVC z-score among Inuit and Caucasians. The mean FEV_1_ z-score (corrected) were −0.2 (95% CI −3.4–4.6) and the mean FVC z-score (corrected) were 0.1 (95% CI −2.9–4.2). This was similar to the mean values for Caucasians; FEV_1_ z-score −0.1 (95% CI −3.7–1.7) and FVC z-score −0.1 (95% CI −1.9–1.9). The association between corrected Inuit lung function and Caucasian lung function were near statistically significant (*p* = 0.05). Male participants classified with probable occupational asthma constituted 4.6% while female participants classified with probable occupational asthma constituted 7.8%. Probable or possible work-related asthma were found among 13.5% of this population with no difference between males and females. Although more females than males were classified with probable occupational rhino conjunctivitis with 8.7% females compared to 3% males, the overall difference was not significantly different (Table 4). Eight participants were classified with both probable occupational asthma and rhino conjunctivitis (2.2%). The mean age for having probable occupational allergy was 37.8 years, for probable occupational asthma 38.6 years while the mean age for those having both diagnoses were 50.5 years. The age difference between the groups was not statistically significant. Participants with work related skin irritation constituted 8.4% with more women (15.2%) than men (5.8%) complaining of skin irritation.

**Table 3:** Sensitization to allergens and lung function measurements.

**Variable**	**Male (♂)**	**Female (♀)**	**Total**	***P*-value for male-female difference**
Atopic^d^, *n* (%)	54 (21.0)	28 (29.2)	82 (23.2)	0.11^a^
Sensitized to Snow crab^e^, *n* (%)	46 (17.9)	18 (18.8)	64 (18.1)	0.85^a^
Sensitized to shrimp^f^, *n* (%)	36 (14.0)	12 (12.5)	48 (13.6)	0.71^a^
Sensitized to fish^g^, *n* (%)	4 (1.9)	1 (1.0)	5 (1.4)	1.00^b^
Sensitized to Anisakis^h^, *n* (%)	82 (34.5)	27 (28.1)	109 (32.6)	0.26^a^
FEV1 z score, mean (95% CI)	0.5 (0.4 to 0.7)	0.5 (0.2 to 0.7)	0.5 (0.4 to 0.6)	0.74^c^
FVC z score, mean (95% CI)	0.8 (0.7 to 1)	0.6 (0.3 to 0.8)	0.8 (0.6 to 0.9)	**0.05** ^c^
FEV1/FVC z score, mean (95% CI)	−0.5 (−0.6 to −0.3)	−0.2 (−0.4 to −0.1)	−0.4 (−0.5 to −0.3)	0.08^c^

a *Pearson Chi^2^-test*.

b *Fisher's exact test*.

c *Independent samples t-test*.

d *Yes, if positive reaction by skin prick test toward a minimum of one common aeroallergen*.

e *Yes, if positive reaction by skin prick test toward raw snow crab, cooked snow crab and in some participants cooking water from snow crab*.

f *Yes, if positive reaction by skin prick test toward raw or cooked Northern Prawn and in some participants cooking water from Northern Prawn*.

g *Yes, if positive reaction by skin prick test toward raw cod or turbot (one participant) or RAST test toward cod (four participants)*.

h *Yes if positive RAST test toward Anisakis simplex. Bold value indicates p < 0.05*.

**Table 4:** Prevalence of occupational asthma and occupational rhino conjunctivitis.

**Variable**	**Male (♂)**	**Female (♀)**	**Total**	***P*-value for male-female difference**
Occupational asthma^b^, *n* (%)				0.66^a^
Probable	12 (4.6)	8 (7.8)	20 (5.5)	
Possible	21 (8.0)	8 (7.8)	29 (8.0)	
Unlikely	46 (17.6)	16 (15.7)	62 (17.1)	
Negative	182 (69.7)	70 (68.6)	252 (69.4)	
Occupational rhino conjunctivitis^c^ *n* (%)				0.10^a^
Probable	8 (3.0)	9 (8.7)	17 (4.6)	
Possible	17 (6.4)	6 (5.8)	23 (6.2)	
Unlikely	32 (12.0)	15 (14.6)	47 (12.7)	
Negative	210 (78.7)	73 (70.9)	283 (76.5)	

a *Pearson Chi^2^-test*.

b *Nineteen missing, of which three are due to lacking information in the questionnaire regarding symptoms from the lower airways at work and sixteen are due to inconclusive results from skin prick tests and/or RAST tests*.

c *Twelve missing, of which nine are due to lacking information in the questionnaire regarding symptoms from upper airways at work and three are due to inconclusive results from skin prick tests and/or RAST tests*.

### Exposure, Sensitization, and Disease

The relative risk (RR) of being sensitized to snow crab if the participants had been exposed to snow crab at work as opposed to those sensitized but not exposed at work was RR = 2.27 (Table 5). For shrimp, fish and *A. simplex*, we did not find a statistically significantly higher risk of being sensitized if exposed to the allergen at work as opposed to those sensitized, but not exposed at work. In total, 108 complained of symptoms from upper or lower airways at work. Of these, 38 were not exposed to snow crab, shrimp, or fish at their present workplace, although seven were sensitized to at least one seafood allergen (snow crab, shrimp, or fish). Their work tasks included administration, packing, landing, cooking in the kitchen and different service tasks in other parts of the factories than the production unit (data not shown). Of the 38 participants, nine were atopic.

**Table 5:** The risk of being sensitized to a specific allergen at work if ever exposed to the allergen at work opposed to participants never exposed to the specific allergen at work.

**Sensitized to allergen at work**	**Worked with allergen source^g^**		**RR (95% CI)**	***P***
	**Yes**	**No**		
Snow crab^c^ (yes/no)	36/133 = 0.27	25/210 = 0.12	**2.27** (1.43–3.61)	**<0.01^a^**
Shrimp^d^ (yes/no)	36/247 = 0.15	11/102 = 0.11	1.35 (0.72–2.55)	0.35^a^
Fish^e^ (yes/no)	5/230 = 0.03	0/125 = 0.0	–	0.17^b^
Anisakis^f^ (yes/no)	75/211 = 0.36	30/112 = 0.27	1.33 (0.93–1.89)	0.11^a^

a *Pearson Chi2 test*.

b *Fisher's exact test*.

c *Yes, if positive reaction by skin prick test toward raw snow crab, cooked snow crab and in some participants cooking water from snow crab*.

d *Yes, if positive reaction by skin prick test toward raw or cooked Northern Prawn and in some participants cooking water from Northern Prawn*.

e *Yes, if positive reaction by skin prick test toward raw cod or turbot (one participant) or RAST test toward cod (four participants)*.

f *Yes, if positive RAST test toward Anisakis simplex*.

g *Yes, if ever worked with snow crab, shrimp, or fish. For Anisakis simplex, yes, if ever worked with fish. Bold value indicates p < 0.05*.

More participants were categorized with probable occupational asthma when currently exposed to snow crab (7.9%) than those currently exposed to shrimp (6.9%). Though, we could not reject that the proportion with probable occupational asthma were alike in the two exposure groups. More participants were categorized with probable occupational rhino conjunctivitis when currently exposed to shrimp (7.8%) than those currently exposed to snow crab (4.3%). Though, we could not reject that the proportion with probable occupational rhino conjunctivitis were alike in the two exposure groups.

**Figure 1:**
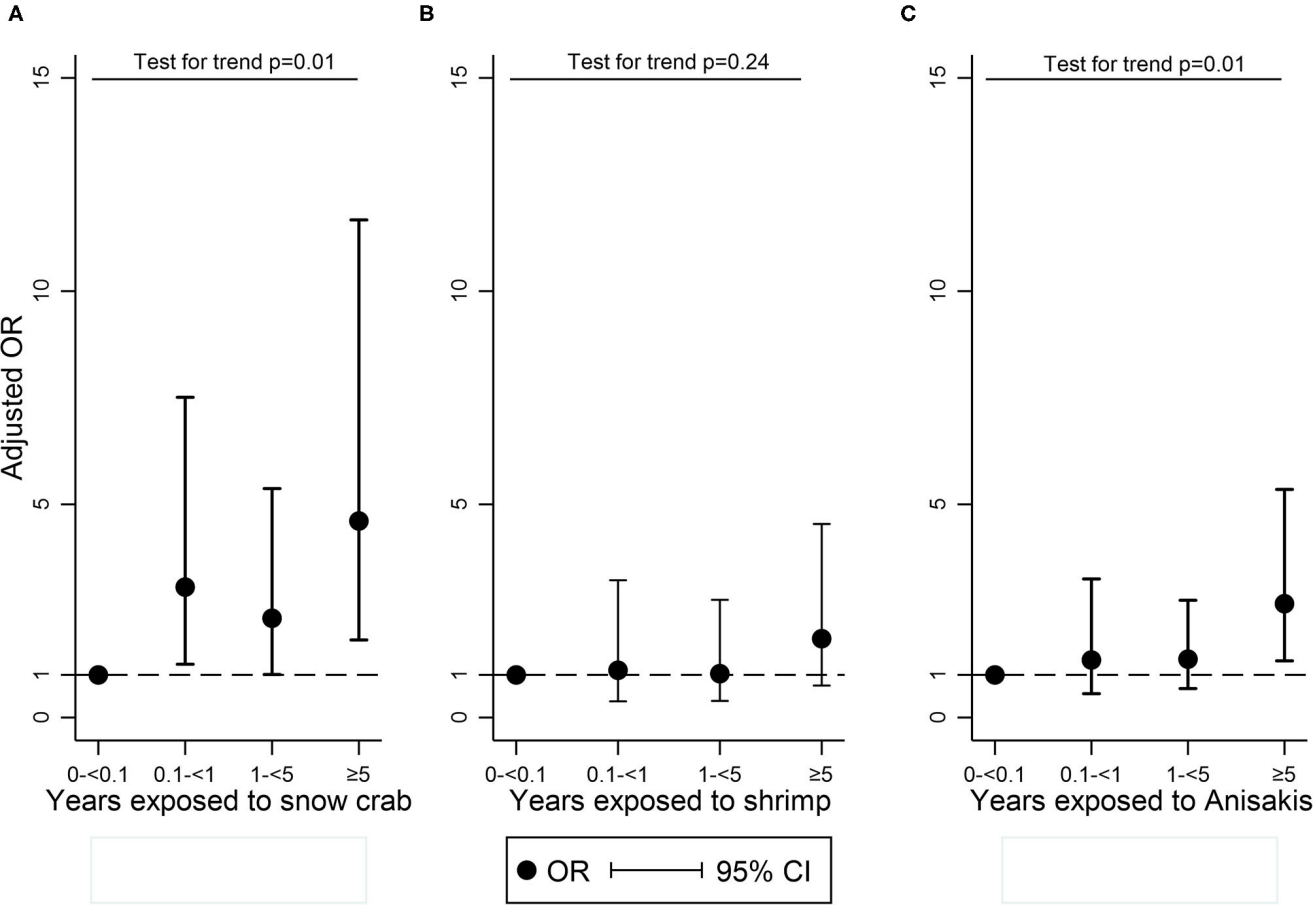
Logistic regression^*^ with trend test exploring a dose-response relationship between sensitization to snow-crab **(A)**, shrimp **(B)**, and *A. simplex* **(C)** and the total time exposed to snow-crab, shrimp, and fish when working in the seafood processing industry in four exposure groups (0–<0.1 year, 0.1–<1 year, 1–<5 years, ≥5 years). The dashed line shows OR = 1.

A potential dose-response relation was analyzed in four exposure groups. The logistic regression analysis was adjusted to atopy, age, smoking, and sex. Increasing duration of exposure to snow crab, compared with 0–<0.1 year of exposure, was associated with increasing odds ratio (OR) of participants being sensitized to snow crab: for 0.1–<1 year, OR = 3.06 (95% CI: 1.25–7.51); for 1–<5 years, OR = 2.33 (95% CI: 1.01–5.37); for ≥5 years, OR = 4.61 (95% CI: 1.82–11.67). The *p* for trend was 0.01 (Figure 1A). Increasing duration of exposure to shrimp, compared with 0–<0.1 year of exposure, was not associated with increasing odds of participants being sensitized to shrimp, and the *p* for trend was 0.24 (Figure 1B). Increasing duration of exposure to fish could not be assessed because of too few sensitized individuals. Increasing duration of exposure to *A. simplex*, compared with 0–<0.1 year of exposure, was associated with increasing odds of participants being sensitized to *A. simplex*: for 0.1–<1 year, OR = 1.35 (95% CI: 0.56-3.25); for 1–<5 years, OR = 1.37 (95% CI: 0.68–2.75); for ≥5 years, OR = 2.67 (95% CI: 1.33–5.35). The *p* for trend was 0.01 (Figure 1C). Furthermore, the trend analysis was stratified to atopy and age in two groups: young (<40 years) and older (>40 years). For snow-crab, the stratification showed that the trend test was only significant among non-atopic and the younger participants. For shrimp, the stratification showed a significant trend among atopic and the older participants. For *A. simplex*, the stratification showed that the trend test was only significant among non-atopic and the older participants.

## Discussion

A considerable proportion of workers in the Greenlandic seafood processing industry had occupational asthma and rhino conjunctivitis and numerous workers were sensitized to occupational allergens.

### Asthma

We found a prevalence of 5.5% for probable occupational asthma. This is higher than the prevalence of asthma among Inuit living in Nuuk (3.6%) (20). Furthermore, the occupational asthma prevalence in the present study is within the range of findings in other studies. Previous studies have found an occupational asthma prevalence ranging from 4 to 36% regarding shellfish and 2 to 8% regarding fish (5, 6, 21). The prevalence range of occupational asthma are widely different in previous studies. This might be due to varying definitions of occupational asthma and the size and the design of previous studies. Many studies include few participants and most of them are cross-sectional (2, 22–27) which may lead to biased results. Furthermore, the type of allergen as well as the type of work process also plays a role (5). As stated earlier by Jeebhay et al., atopy increases the risk of developing occupational asthma (28). In the present study, 23% were atopic representing a risk factor for occupational asthma in the future, which is supported by our finding of a higher proportion sensitized toward snow crab among atopics vs. non-atopics. Of the participants, 90% were smokers or former smokers. Smoking has been mentioned as a risk factor for developing occupational asthma among crab processing workers (28), but a new, large Danish study have indicated that smoking is a protective factor in having asthma (29). In the present study, the number of never smokers was so small that it did not make sense to examine possible differences in the risk of asthma between smokers and never smokers. Finally, we investigated if there was a difference in occupational asthma prevalence among those exposed to snow crab to those exposed to shrimp at their present workplace. We did not find a significant difference between these two groups. More participants were sensitized to snow crab than to shrimp allergens despite higher exposure levels in the shrimp production compared to the snow crab production, both regarding particles, endotoxin, and protein levels (Birgitte Hamann Laustsen et al., Work environment, occupational diseases, and accidents among seafood industry workers in Greenland, submitted). Based on this contradiction, one could suspect snow crab allergens to be more potent in inducing sensitization, asthma, and allergy than shrimp allergens. Though, the most likely explanation for this contradiction may be that snow crab workers were more exposed to allergens due to their working conditions than shrimp workers. In the present study, 108 participants complained of symptoms from the upper or lower airways at work. Of these, 38 participants were not exposed to snow crab, shrimp, or fish at their present workplace but seven were sensitized to snow crab, shrimp, and/or fish. These seven participants may have been exposed and sensitized at earlier workplaces or in their leisure time. We know from previous studies that kitchen cooks and caterers are also at risk of being sensitized when handling crustaceans and fish (30, 31). However, only two participants worked as kitchen cooks and only one of them complained of symptoms. Nine of the 38 participants were atopic, which may also explain their symptoms. In addition, former studies have shown that not only seafood allergens cause allergic/inflammatory reactions. Also other work-related exposures, such as endotoxins, bacteria, viruses, parasites, marine or bacterial toxins, gases, protochordates, algae, chemical additives, spices, and hidden ingredients in canned or processed fish products can cause symptoms from upper and lower airways at work (28, 32), which might explain these symptoms among those not exposed to the specific allergens we studied.

### Rhino Conjunctivitis

We found a prevalence of 4.6% for probable occupational rhino conjunctivitis. Previous studies have found an occupational rhinitis prevalence of 5–24% (5). Often, occupational rhino conjunctivitis and occupational asthma has been found in the same individuals. In fact, occupational rhino conjunctivitis tends to present before occupational asthma in the same individuals (5, 33). Therefore, occupational rhino conjunctivitis may be an indicator of underlying allergic disease posing a higher risk of also developing occupational asthma (28). In other words, in addition to the 5.5% categorized with probable occupational asthma, the 2.5% participants categorized with only probable occupational rhino conjunctivitis are also at risk of developing occupational asthma. In the present study we found eight participants with both probable occupational asthma and rhino conjunctivitis equalling 40% of all categorized with probable occupational asthma and 47% of those with occupational rhino conjunctivitis. We found no significant difference in the prevalence of occupational rhino conjunctivitis among those exposed to snow crab and those exposed to shrimp at their present workplace.

### Sensitization to Snow Crab, Shrimp, and Fish

It has been known for decades that the mechanism for sensitization toward airborne seafood allergens at work is mostly IgE mediated (2, 7). Later studies have shown that especially high molecular weight proteins are involved in the pathogenesis (8). In the present study, we found high sensitization levels to specific seafood derived allergens. The highest frequency of IgE sensitization were identified toward snow crab (18.1%) followed by shrimp (13.6%) and fish (1.4%). Studies in the seafood processing industry in other parts of the world have observed similar results with sensitization levels ranging from 13.6 to 25% to snow crab (4, 34–36), 12.5 to 24% to shrimp (3, 22–24), and 1.6 to 8.2% to fish (3, 22, 37). In general, IgE sensitization to shellfish-allergens are more prevalent than fish-allergens, which is also found in the present study (6, 32, 38). Possibly the cooking and vapor exposure of the shellfish in contrast to the handling of raw fish also contributes to the higher risk of sensitization when processing shellfish (5). Our exposure measurements showed the highest exposure levels in the shrimp production (Birgitte Hamann Laustsen et al., Work environment, occupational diseases, and accidents among seafood industry workers in Greenland, submitted). Thus, the observation of the highest sensitization levels toward snow crab is surprising. These findings seem to indicate that snow crab allergens are more allergenic than shrimp allergens through inhalational exposure and sensitization. Furthermore, based on our understanding that allergen-specific IgE sensitization leads to a higher risk of developing allergy, which further leads to a higher risk of developing asthma, it seems that snow crab allergens may be more likely to cause allergy and asthma than shrimp allergens (5, 28, 33). In the present study, we found a dose-response effect between exposure to snow crab at the current workplace and the risk of being sensitized to snow crab. We did not find a dose-response effect for shrimp and it was not possible to examine the association for fish due to too few fish-sensitized participants. The lack of a dose-response effect for shrimp might be because participants are frequently exposed to shrimp allergens in their leisure time as shrimp are commonly consumed in Greenland in contrast to snow crab. We do not, however, have data on the non-occupational exposure of the participants to seafood, e.g., by consumption of seafood, and cannot distinguish between sensitization acquired at or outside work. Furthermore, there might be a healthy worker effect with workers leaving their jobs soon after debut of symptoms. For shrimp, the stratification on age showed that the trend test was significant for those above 40 years. This is most likely because the older participants ingest more traditional food including shrimp. A dose–response relationship between the dose of seafood and the risk of developing sensitization or occupational asthma have been found in earlier studies (5, 28, 39). Furthermore, Hudson et al. found that the prognosis for recovery from seafood mediated airway disease worsened, the longer the exposure to seafood extended over time (40). Malo et al. found that after 2 years of termination of seafood exposure, remission of occupational asthma reached a plateau (41). We found a RR of 2.3 of being sensitized to snow crab if the participants had ever worked with snow crab as opposed to participants never exposed to snow crab at work. We did not find a significantly higher risk of being sensitized to shrimp or fish if ever working with shrimp or fish. Again, this might be explained by differences in leisure time activities and food ingestion.

### Sensitization to *Anisakis simplex*

A sensitization prevalence towards *A. simplex* of 32.6% in the present study is consistent with findings in other studies varying from 1.8 to 50% (13, 14). Our findings do not allow us to distinguish between *A. simplex* sensitization due to ingestion of seafood or due to occupational exposure. An important difference between our study and former studies is the cut off value of 0.1 kU/L while in former studies a value 0.35 kU/L was used. The lower cut-off was used because ImmunoCAP has improved the sensitivity of their quantification in recent years (42, 43). All values between 0.1 and 0.35 kU/L are considered true positives. However, if applying the higher cut off value, the number of sensitized workers declined only marginally to 26%, while only 0.5% were sensitized to fish. To the authors knowledge, this is the first study to show a significant dose–response relationship between exposure-time to fish at the workplace and the risk of sensitization to *A. simplex*. For *A. simplex*, the stratification on age showed that the trend test was significant for those above 40 years. This is most likely because the older participants ingest more traditional food including fish. Indeed, *A. simplex* is known to show extremely high sensitization levels in the general population (13). We did not find a significantly higher risk of being sensitized to *A. simplex* if ever working with fish supporting the theory that *A. simplex* sensitization is partly due to consuming fish rather than being exposed to *A. simplex* at work. Moreover, a recent study showed that the highest risk associated with *A. simplex*-sensitization among workers was fishing in their leisure time, rather than any of attributes related to the occupational exposure (14).

### Lung Function

Since no Inuit reference material exists for lung function measurements, we were constrained to use the Caucasian reference material as suggested by Fenton et al. (44). Lung function measurements assessed by FEV_1_ and FVC were higher than expected with both FEV_1_ z-score and FVC z-score above zero. The z-scores for FEV_1_ and FVC showed some low values with a shift to the top range as well. Inuit lung function has previously been demonstrated to be higher than Caucasian lung function (17). Hence, the reason for higher lung function among the participants may be genetical. We tried to do a standing/sitting height correction of all participants with Inuit ethnicity with the ratio 0.54/0.52 due to former studies showing that Inuit have shorter legs than Caucasians (45, 46). This led to similar FEV_1_ z-score and FVC z-score among Inuit and Caucasians. However, the higher lung function in the present study may also partly be explained by a healthy worker effect in which the workers with the lowest lung function quit their jobs in the seafood processing industry shortly after employment began.

### Skin Symptoms

In the present study, we found more females than males complaining of symptoms from the skin. This may be due to different work tasks among males and females. Males were more likely to be engaged in service, handling and packing tasks while females were more likely to be engaged in fish and shrimp handling and lab work. Hence, females may be more exposed to wet work and gloves which irritates the skin. The difference between males and females may also be explained by a difference in gender's attitude toward health problems (47).

### Strengths

Considerable strengths of the present study are the size of the study and the high participation rate, although the number of eligible workers could not be precisely determined. Of the invited workers, 84.5% participated. Of the workers present at the factories on the examination days, nearly 100% participated. A great effort was done in order to overcome language barriers. The questionnaire was in both Danish and Greenlandic. If the participants had problems understanding the questions, Danish-Greenlandic medical students conducted interviews.

### Weaknesses

The gold standard for diagnosing occupational asthma in epidemiological studies is a questionnaire and allergy test (skin prick test or RAST) followed by specific bronchial provocation (48) with serial peak flow measurements at and away from work being almost as valuable in many cases. Unfortunately, in the present study it was not possible to perform specific bronchial provocations or serial peak flow measurements due to constraints on time and compliance problems. Therefore, the diagnosis of occupational asthma relied on answers regarding symptoms from the lower airways at work in the questionnaire, lung function measurements, reversibility testing, and allergy tests toward work allergens. Hence, the diagnosis of occupational asthma possesses some uncertainty in the present study, and we may not have diagnosed everyone with occupational asthma. On the contrary, we may have diagnosed some with asthma who instead suffered from chronic obstructive pulmonary disease (COPD). In general, the risk estimates are probably conservative because there is a high risk of misclassification in our exposure data, possibly decreasing the likelihood of detecting associations. In assessing occupational exposure, we did not have access to complete job histories from employers and we relied on the participants subjective memories of past and present jobs including time frame and tasks. Although non-differential misclassification most often occurs in association with subjective exposure assessment, differential misclassification cannot completely be ruled out due to a possible common knowledge in the communities of an association between the present exposure and disease thus intending to overestimate the risk. Since it was not possible to establish contact with the last 15% of the employees, a potential risk of selection bias may exist. However, due to the size of the study, the modest loss of 15% and the general homogeneity of the employees in this specific sector in Greenland, we expect bias to a limited extent. Due to the possibility of misclassification discussed above, the data should be considered with care. Further studies should include a prospective follow-up design in order to identify causal relations, to evaluate how symptoms evolve among the workers, and to identify potential preventive measures.

## Conclusion

This the first study regarding occupational asthma and rhino conjunctivitis in the Greenlandic seafood processing industry. The study showed that a considerable proportion of workers in the Greenlandic seafood processing industry had occupational asthma and rhino conjunctivitis. This supports the hypothesis of a considerable degree of underreporting of occupational allergic airway disease in the Greenlandic seafood processing industry. This may be caused by a lack of knowledge of the reporting system both among employees, employers, and health professionals. Furthermore, we found high sensitization levels toward snow crab, shrimp, and the fish parasite, *A. simplex*. Finally, we found a dose–response relationship between the risk of being sensitized to snow crab and *A. simplex* according to work length, though *A. simplex* sensitization may partly be caused by ingestion of fish and crustaceans. A large proportion of the participants were atopic and thereby possess a considerable risk of developing allergic airway disease in the future if an ongoing exposure to seafood allergens is maintained. Prospectively, it is important to inform workers, leaders, and health care professionals of the health problems and the law on worker's compensation.

## Data Availability Statement

The datasets presented in this article are not readily available because the authors do not have permission to share the data. Requests to access the datasets should be directed to birgitte.l@rn.dk.

## Ethics Statement

The studies involving human participants were reviewed and approved by the Danish Data Protection Agency, the Central Denmark Region, The Scientific Ethical Committee for Greenland (2015-11317) and the Human Research Ethics Committee for James Cook University, Australia (H8114). Written informed consent for participation was not provided by the participants' legal guardians/next of kin because: Had we been aware beforehand that a few workers were children aged 16 or 17 years we would have applied for research ethics approval of including them using just their own signature and acceptance in accordance with Danish research ethics requirements for children not exposed to any medical interventions in scientific studies. However, we were not aware in time, and regret to admit that we did not do that. Subsequently, The Scientific Ethical Committee for Greenland has been notified and has approved this issue.

## Author Contributions

BL, JB, TS, NE, and KR designed the study. BL drafted the manuscript and performed the statistical analysis with contribution from ØO, EW, TS, NE, OC, KR, SK, AL, and JB. All authors interpreted and discussed the results. ØO, TS, NE, OC, KR, and JB acquired the data. All authors contributed to revising the manuscript critically and approved the final draft for publication.

## Funding

This work was supported by the Danish Working Environment Research Fund (grant number 20165103740), The Health Science Research Council, The Work Environment Council of Greenland, the Greenlandic Workers' Union, Greenland Business Association, Royal Greenland, Polar Seafood, Bank of Greenland. SK received a Peter Doherty Research Fellowship from the National Health and Medical Research Council, Australia (GNT1124143).

## Conflict of Interest

The authors declare that the research was conducted in the absence of any commercial or financial relationships that could be construed as a potential conflict of interest.

## Publisher's Note

All claims expressed in this article are solely those of the authors and do not necessarily represent those of their affiliated organizations, or those of the publisher, the editors and the reviewers. Any product that may be evaluated in this article, or claim that may be made by its manufacturer, is not guaranteed or endorsed by the publisher.

## Footnotes

^1^*ECRHS II Lung Function Protocol and Questionnaire*. Available online at: https://www.ecrhs.org/questionnaires-and-protocols (accessed September 7, 2021).

^2^*SPSS Macro*. Available online at: https://dev.ers-education.org/lr/show-details/?idParent=138989 (accessed September 18, 2020).

^3^IBM. Available online at: www.ibm.com (accessed September 18, 2020).
